# Ethnobotanical assessment of plant resources of Banda Daud Shah, District Karak, Pakistan

**DOI:** 10.1186/1746-4269-9-77

**Published:** 2013-11-22

**Authors:** Waheed Murad, Azizullah Azizullah, Muhammad Adnan, Akash Tariq, Kalim Ullah Khan, Saqib Waheed, Ashfaq Ahmad

**Affiliations:** 1 Department of Botany, Kohat University of Science and Technology, Kohat 26000, Pakistan; 2 Department of Botany, Islamia College University Peshawar, Peshawar 25000, Pakistan

**Keywords:** Indigenous knowledge, Ethnomedicinal uses, Livelihood, Conservation

## Abstract

**Background:**

The Indigenous knowledge of plants is scientifically and culturally very significant. This paper elucidates the empirical findings of an ethnobotanical survey of Banda Daud Shah, District Karak, Pakistan.

**Methods:**

Data collection was carried out from October 2011 to September 2012. Total twelve survey trips were made, three in each season. About 100 respondents were interviewed; most of them were aged people between 60–70 years. Interviews were conducted using structured questionnaire composed of variety of questions regarding ethnomedicinal uses of plants of the study area. Direct matrix ranking (DMR), informant citations and market survey of multipurpose plants were also carried out.

**Results:**

The local community was using 58 plant species belonging to 52 genera and 34 families for different purposes. A total of 25 plant species were herbs followed by 18 shrubs. Leaf (45%) was the most commonly used plant part followed by the whole plants (23%). In total, 40 plant species were medicinally used to treat variety of diseases, of which highest number of species being used for gastro-intestinal problems (19 spp.), expectorant (3 spp.) and antipyretic (3 spp.). Beside medicinal values, 25 species were used for fuel and 18 for fodder purposes. Informant consensus showed that gastrointestinal and respiratory infections were ranked highest (F_IC_ = 0.75) among all ailments. According to DMR output, *Dalbergia sisso* ranked first due to high multipurpose uses among all species and was found most threatened with higher market value.

**Conclusion:**

The investigated area is rural in nature and the inhabitants are highly dependent on the native plants for their health care needs and other requirements like fuel wood and fodder due to financial constraints and unavailability of resources. Medicinal plants for high ranked diseases may be phtyochemicaly and pharmacologically investigated to prove their efficacy. The local medicinal flora is facing overexploitation, overgrazing and improper way of collection. Proper conservation strategies such as controlled grazing, reforestation and rangeland management among many others may be adopted to promote the sustainable use of medicinal plants.

## Introduction

Plants are a vital source of traditional medicines that are used for the treatment of various ailments [1]. Approximately 4, 22,000 flowering plants reported from the world, more than 50,000 have been used worldwide for medicinal purposes [2]. Main objective of ethnobotanical research is to record the indigenous uses of plant resources. Until now, 80% of the world’s population depends on traditional medicines for its primary health care needs [3]. Plants remedies are often used as an alternative to allopathic medicines [4]. Inhabitants of the remote areas have good knowledge about the utilization of plants. Local people prefer medicinal plants due to their easy availability and cheap therapy as compared to costly pharmaceuticals. Local people have discovered the therapeutic activity of medicinal plants against certain diseases through their indigenous experiences transferred to them from their ancestors [5]. Recently, the field of ethnobotany has shown tendency from mere documentation process to a more practical one, emphasizing on the sustainable use of local medicinal flora. Treatment through traditional medicine system is progressing all over the world particularly in the Indian sub-contentment where this system is known by Unani or Ayurvedic [6].

Pakistan is bestowed with a unique biodiversity that is stretched along nine major ecological zones. A major part of country is quite rich in medicinal herbs due to its salubrious climate [7]. Numerous ethnomedicinal plants extracted from the wild are being used by local communities of different geographical regions having century’s old traditional knowledge on such plants. A number of medicinal plants are well known about their properties and proper use at the community level, however many are yet to be explored for their medicinal values [8]. In early 1950 more than 80% of Pakistani population was totally dependent on ethnomedicines for traditional health practices [9], but now it is experienced only in the rural areas [10], because the indigenous knowledge develops and changes with the passage of time, with change of natural resources and culture. Indigenous knowledge on ethnomedicines is under threat due to the current modernizing trends among the rural societies, which is reducing the locals’ wisdom on the precious flora. In Pakistan, about 6000 plant species have been reported so far, however ethnomedicinal knowledge on 600 plant species only have been documented [11]. There is a dire need to preserve this valuable traditional knowledge [12].

Preserving and promoting indigenous knowledge on medicinal plants are considered as rescuing the global heritage in addition to the new drugs discovery [13]. High priority should be given to the documentation of indigenous knowledge on the plants’ ethnomedicinal use in order to ensure their protection for the present and future generation [14]. The present study was conducted in Banda Daud Shah, District Karak where agriculture is negligible and hence most people are dependent on the wild sources for different ethnobotanical purposes. Many studies have been conducted on the indigenous uses of medicinal plants in Pakistan [15-23] but investigated area is still unexplored. Therefore, a need was felt to document and conserve the traditional knowledge of the area before the information is lost forever. Hence, the present study aimed to explore the traditional knowledge, possible threats and conservation strategies with respect to the local flora. In addition, the study also aimed to select candidate medicinal plant species of high informants’ consensus value. Specific objectives are (i) to identify medicinal plants and explore their uses as non timber forest products (NTFPs) (ii) to investigate the indigenous knowledge on ethnomedicines, and (iii) to assess the plants conservation issues of the study area.

## Materials and methods

### Study area

**Figure 1:**
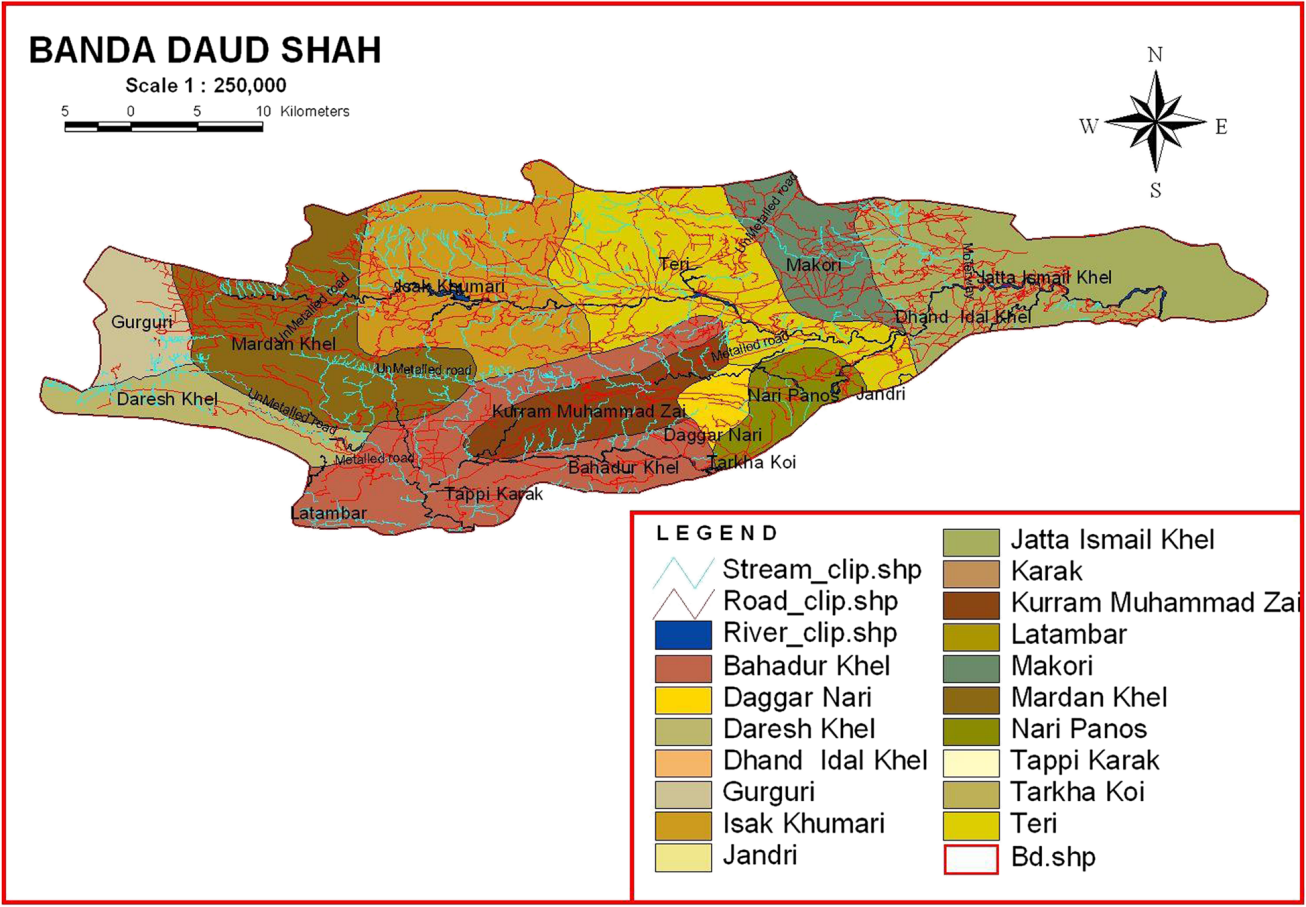
Map of the study area.

Banda Daud Shah has a total area of 600 Km^2^ and lies in the south of Khyber Pakhtunkhwa Province between 70-40° to 71-30° N latitude and 32-48° to 33-23° E longitude [14]. It is bounded in the North-West by District Hangu, in the North-East by District Kohat, in the South-West by Tribal area and in the South by Karak Tehsil (Figure 1). The study area is divided into mountainous area, small hills and plains having most of clayey or sandy soil. Wheat, corn and gram are the common cultivated crops. Various plant species dominated the study area such as *Acacia modesta, Acacia nilotica, Adhatoda vasica, Aerva javanica, Dodonaea viscosa, Eucalyptus lanceolatus, Fagonia cretica, Rhazya stricta, Saccharum arundinaceum, Withania coagulans and Withania somnifera*.

### Informant selection

General information about the study area was collected before start of the research work. Medicinal plants grow in different seasons and therefore, ethnobotanical data was collected during summer, autumn, spring and winter from October 2011 to September 2012. Data was collected in twelve survey trips i.e. three in each season. Data collection and voucher specimens were collected following standard method of Martin [24]. Informants’ sample size was selected on the basis of information provided by the local administrative officers. In total 100 native informants were selected as resource persons for data collection. Among all the informants, 70 were old age people (52 males, 18 females), which were aged between 50–70 years. Whereas, ages of 19 informants ranged between 30–45 years, in which mostly females (13) were interviewed. Remaining 11 informants (6 males, 5 females) were below 30 years of age. The selected respondents were experts in field of medicinal plants and were having great reputation in the society about their knowledge on traditional medicines. These informants were social workers, farmers, rural herbalists (*Hakeem’s*) and housewives. Before data collection, a brief group discussion was held with the key informants, in which objectives of research were explained to them. This was done in order to acknowledge informants’ cooperation in preserving the traditional knowledge of the study area and build their confidence for providing reliable information.

### Data collection, organization and analysis

Structured questionnaire was designed containing predefined questions about medicinal plants. Informants were asked about the local names of plants, ailment treated, part used, other uses beside medicinal, most commonly used plants. In addition, focus group discussions were also designed so as to gain further information on medicinal plants knowledge of the community and prove the reliability of the data collected through questionnaire. Interviews were conducted in the local language (*Pashto*) by visiting each respondent individually. The habits of the studied plants were documented from the online literature [25]. Plant parts were classified into 8 categories like leaves, roots, whole plant etc. Other uses besides medicinal uses were grouped into 11 categories like fuel wood, sheltering plants, fodder, agricultural tools etc. Habits of the plants were categorized into three parts like herbs, shrubs and trees.

Data on use diversity of multipurpose medicinal plants were evaluated by a direct matrix ranking (DMR) exercises as described in Cotton [26] that involved fifteen (ten men and five women) key informants. Participants for this exercise were selected based on their long years of experience as traditional herbal practitioners in the study area as described in Yineger *et al*. [27]. Market survey was also conducted for multipurpose medicinal plants. Market value of medicinal plants was recorded as US$ Kg^-1^ for prices uniformity.

Informant consensus factor (F_IC_) was computed after the reported traditional remedies and corresponding diseases were grouped into 12 categories. F_IC_ was obtained by computing number of use citations in each disease category (Nur) minus the number of times a species used (Nt), divided by the number of use citations in each category minus one [28].

ICF=Nur‒Nt/Nur‒1

### Preservation of plants

The plants were pressed, dried and mounted on the herbarium sheets. Plants were identified by the expert taxonomists at the Department of Botany, Kohat University of Science and Technology. Scientific names (Latin), family names and names of publication authors were corrected according to the Flora of Pakistan [25]. For confirmation, the studied plants samples were compared with the already identified plant specimens preserved in the herbarium of University of Peshawar, Pakistan. The correctly identified specimens were deposited in the herbarium, Department of Botany, Kohat University of Science and Technology, Kohat.

## Results

**Figure 2:**
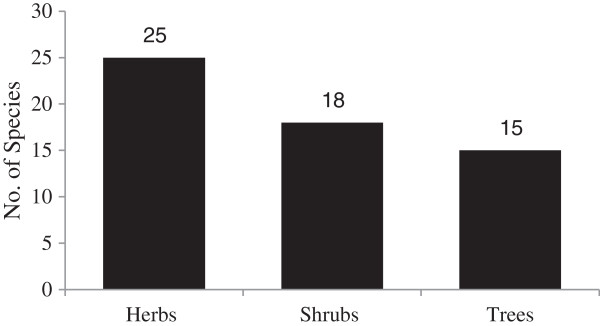
Classification of plants on the basis of their habits.

**Figure 3:**
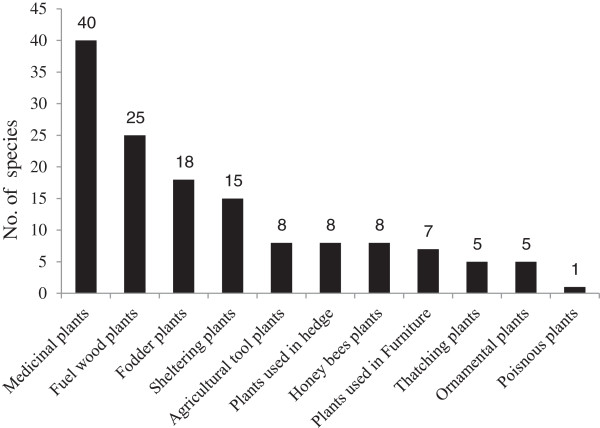
Non timber forest products and uses.

**Figure 4:**
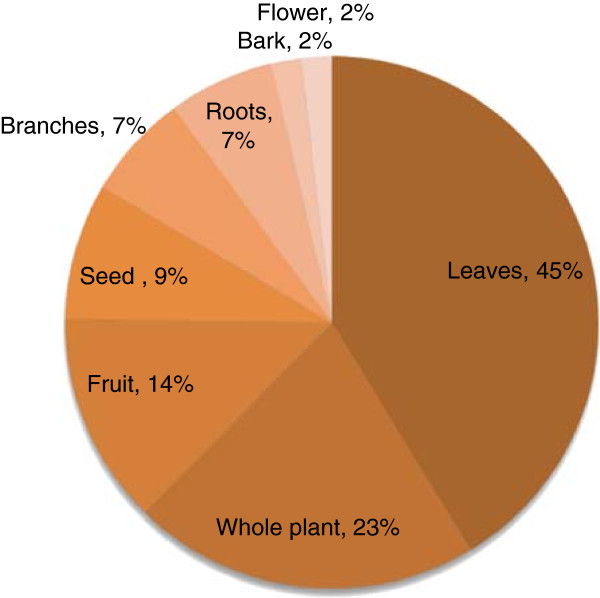
Percentage of plant parts used in traditional medicines.

**Figure 5:**
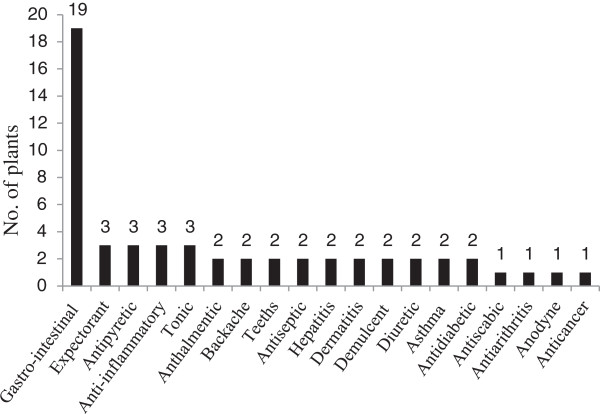
Number of medicinal plants used for various human ailments.

A total of 58 plant species belonging to 52 genera and 34 families were collected from the study area. All the species were identified and their ethnobotanical information was elucidated (Table 1). Out of 34 families, 3 were monocotyledons (Arecaceae, Poaceae and Typhaceae) and the remaining 31 families belonged to dicotyledons. The dominant family was Solanaceae (5 spp.), followed by Asclepiadaceae, Asteraceae, Brassicaceae, Mimosaceae, Papalionaceae and Poaceae each were sharing 3 species (Table 1). Out of total plant species, 25 were herbs followed by 18 shrubs species (Figure 2). Moreover, out of the total 58 plant species, 40 were used for the preparation of various ethnomedicines, 25 as fuel wood and 18 were used as fodder species (Figure 3). Mostly the leaves of 45% plant species were used for medicinal purpose followed by 23% species used as a whole plant (Figure 4). Out of the 40 medicinal plants, 19 were used against gastro-intestinal disorders followed by 3 each as expectorant and antipyretic (Figure 5). The output of the DMR exercise on ten multipurpose medicinal plants enabled to identify which of the multipurpose plants is most under pressure in the area and the corresponding factors that threaten the plant. Accordingly, *Dalbergia sisso* ranked first (most threatened); *Acacia modesta* and *Acacia nilotica* ranked second; *Morus nigra* ranked third; *Ziziphus nummularia* ranked fourth, while *Monotheca buxifolia* and *Capparis desidua* ranked fifth (Table 2). Results also indicated that those multipurpose medicinal plant species are currently exploited more for construction, firewood and fodder purposes than for their medicinal role. Market survey showed that among tree species, *Dalbergia sissoo* ranked first with higher market price (0.6 US$ Kg^-1^) (Table 2). Table 3 showed the informant consensus factor (F_IC_) for different ailments treated by traditional healers in the study area. The level of informants agreement was high for most ailment categories (mean F_IC_ = 0.75) like gastrointestinal and respiratory infections and total consensus (F_IC_ = 1.00) was even obtained for dermatological, inflammations, cardiovascular, fever and dental problems. Results also showed that no ailments have low level of consensus.

**Table 1:** Ethnobotany of Banda Daud Shah, District Karak

**Plant families**	**Plant species**	**Local names**	**Habit**	**Part use**	**Ethnobotanical use**
Acanthaceae	*Justicia adhatoda* Linn. KUH-129	Baza	Shrub	Leaves	The leaves are crushed and used as expectorant. It is also used as anti-inflammatory agent. The plant attracts honey bees.
Amaranthaceae	*Aerva javanica* (Burm. f.) Juss. KUH-103	Khursasa	Shrub	Whole plant	The plant is boiled in water and used as demulcent and diuretic. Dried plant is used as fuel.
	*Amaranthus viridis* Linn. KUH-106	Ranzaka	Herb	Leaves	The leaves are used for gastro-intestinal problems. It is used as fodder.
Apocynaceae	Nerium oleander Linn. KUH-138	Gandiray	Shrub	Whole plant	It is cultivated for ornamental purposes.
	*Rhazya stricta* Dcne. KUH-147	Gandarai	Shrub	Root and leaves	The Leaves extract is used as anticancer. Extract of root is used for toothache. Leaves are placed in wheat seeds to protect from insect. Dried leaves is also used as a fuel, hedge and sheltering. It is also honey bee species.
Arecaceae	*Nannorrhops ritchieana* Griff. KUH-137	Mazaray	Herb	Leaves	The decoction of leaves is used for stomach problems. It is also used as thatches.
	*Phoenix dactylifera* Linn. KUH-144	Khajora	Tree	Whole plant	Fruit is laxative. Leaves are used for making mates, ropes and basket.
Asclepiadaceae	Aloe vera Linn. KUH-105	Zargia	Herb	Leaves	The decoction of leaves is used as anti-arthritis and backache. Extracts of leaves is used for hepatitis and dermatitis.
	*Asphodelous tenuifolius* Cavan. KUH-107	Pyazakay	Herb	Leaves and seeds	Leaves are said to be diuretic. Seeds are externally applied to ulcers and inflamed parts by local people. It is also a honey bee’s species.
	*Calotropis procera* (Wild) R. Br. KUH-112	Spalmaka	Shrub	Whole plant	Dried leaves are crushed and used as expectorant and anthelmentic. Young flowers used for tumors. The whole plant is used as fodder and fuel
Asteraceae	*Calendula arvensis* Linn. KUH-110	Zirgulay	Herb	Leaves	Leaves are used for hepatitis and spleen enlargement control
	Launaea nudicaulis. KUH-132	Peshtlari	Herb	Leaves	The paste of leaves is used for wound healing and antiseptic.
	*Parthenium hysterophorus* Linn. KUH-142	Livanay banga	Herb	Leaves and branches	Used as fodder and fuel.
Boraginaceae	*Heliotropium bacciferum* Forrsk. KUH-128	Markondi	Herb	Seed	Seed is ground and used for backache.
Brassicaceae	*Brassica campestris* Linn. KUH-109	Oray	Shrub	Whole plant	The plant is used as fodder and fuel.
	*Eruca sativa* Mill. KUH-121	Sharsham	Herb	Whole plant	Oil extracted from seed used as anti-lice agent, anti-inflammatory and anti-scabic. It is fodder and fuel plant.
	*Sisymbrium irio* Linn. KUH-150	Shonopy	Herb	Seed and leaves	Leaves are used for stomach problems. Leaves are used to as antipyretic. Seed are used as anti vomiting.
Cactaceae	*Opuntia dillinni*. KUH-140	Papar	Shrub	Whole plant	It is cultivated at the border of crop field to protect them from grazing animal.
Capparidaceae	*Cappris decidua* Forssk. KUH-113	Kirra	Tree	Whole plant	Fruit is laxative used in pickles and jams. Bark is anthelmentic and used for swellings. It is honey bees specie. The pulp of the ripened fruit is edible. The plant is used for hedging and sheltering. Dry plant is used for fuel and making agriculture tools.
Celasteracea	*Gymnosporia royleana* Wall. KUH-127	Hrazanka	Shrub	Leaves	The leaves are used as fuel and fodder.
Chenopodiaceae	*Chenopodium murale* Linn. KUH-114	Sorma	Herb	Leaves	The plant is used as a fodder.
	Kochia indica Wight. KUH-131	Qurashka	Herb	Whole plant	Dried plant is used as fuel.
Convolvulaceae	*Convolvulus arvensis* Linn. KUH-115	Parwata	Herb	Whole plant	The root is laxative. The plant is used for skin diseases and asthma. It is a honey bee species and also used as fodder.
Euphorbiaceae	*Euphorbia helioscopia Linn. KUH-123*	Zarobotay	Herb	Whole plant	Poisonous, excessive use of plant causes death of cattle.
	*Euphorbia exigua* Linn. KUH-124	Botay	Herb	Whole plant	The plant serves as a fodder.
Fumariaceae	*Fumaria indica* Hausskn. KUH-126	Livanay gajara	Herb	Whole plant	The extract of plant is used as antipyretic and anthalmentic.
Lamiaceae	*Otostegia limbata* (Benth.) Boiss. KUH-141	Spinazghi	Shrub	Whole plant	It is used as fuel, hedge and sheltering.
Malvaceae	*Malva parviflora* Linn. KUH-133	Puskay	Herb	Leaves	The decoctions of leaves are used for stomach problem. It is also used as laxative.
Meliaceae	*Melia azedarach* Linn. KUH-134	Bakanra	Tree	Leaves and seed	Leaves are antiseptic. Seeds are used for blood pressure. The plant is used for ornamental purposes, fuel, shelter and furniture.
Mimosaceae	Acacia modesta Wall. KUH-101	Palosa	Tree	Whole plant	Leaves act as cooling agent. Bark act as pain killer. It is honey bees species. Root and stem are used for making furniture and agriculture tools. It is also used as hedging and shelter.
	*Acacia nilotica* Linn. KUH-102	Kikar	Tree	Whole plant	Bark is used for stomach problems. Seed is demulcent and stimulant. The plant is used as fodder, fuel, agricultural tools and furniture.
	*Prosopus juliflora* (Sw) DC. KUH-145	Kikray	Tree	Branches	Branches are used for hedging and sheltering and also for fuel and fodder.
Moraceae	*Morus nigra* Linn. KUH-136	Toot	Tree	Wood and fruit	Wood is used for fuel, furniture and agriculture tools. Fruit is eaten.
Myrtaceae	*Callistemon lanceolatus* Sm. KUH-111	Bottle bursh	Tree	Whole plant	It is cultivated for ornamental purposes.
	*Eucalyptus lanceolatus* Linn. KUH-122	Lochay	Tree	Branches and fruit	Fruit is laxative and digestive. Branches are used for thatching, hedging and sheltering.
Oleaceae	*Olea cuspidata* Wall. KUH-139	Zaitoon	Tree	Leaves and fruit	Leaves are antiseptic. Fruit are use as tonic. The plant is used for furniture and agriculture tools.
Papilionaceae	*Albizia lebback* Linn. KUH-104	Sreen	Tree	Branches	The branches are used for fuel and shelter. It also serves as furniture making plant.
	*Astragulus tragacantha* Linn. KUH-108	Warikay hrazana	Herb	Leaves	Leaves are used for respiratory infection. It is a fuel and fodder plant.
	*Dalbergia sissoo* Roxb. KUH-118	Shawa	Tree	Whole plant	The plant is used for fuel, agricultural tools, furniture and sheltering purposes.
Poaceae	*Cymbopogan jwarancusa* (jones) Schult. KUH-116	Sargaray	Herb	Whole plant	The plant is tonic and it is also used as fodder and fuel.
	*Cynodon dactylon* (L) Pers. KUH-117	Osha	Herb	Whole plant	Leaves are laxative and are used in asthma. Whole plant is used as a fodder and for ornamental purposes.
	*Saccharum arundinaceae* Hook. KUH-148	Muskanray	Shrub	Leaves	Used for fuel and fodder. It also used in making roof of houses.
Punicaceae	*Punica granatum* Linn. KUH-146	Anar	Tree	Fruit and bark	Fruit is eaten which compensate iron deficiency. Bark is used for nasal congestion.
Rhamnaceae	*Zizyphus jujuba* Mill. KUH-157	Bera	Tree	Whole plant	Fruit is laxative. Fruit is eaten and also use in medicine. It is honey bees specie. The plant is used as a fuel and fodder. It is also used for making agriculture tools.
	*Zizyphus nummularia* (Burm. f.) KUH-158	Karkanra	Tree	Whole plant	Medicinally the fruit is tonic and digestive. Young leaves are use as anti-diabetic. Ripened fruit is eaten. Dry plant is used as fuel and for agriculture tools. Branches are use for hedging and fencing.
Sapindaceae	*Dodonaea viscosa* Linn. KUH-120	Zerawana	Shrub	Leaves and branches	Leaves are astringent and used in goat rheumatism. Branches are used in thatching and hedging. It also serves as a fodder. Dried plant is used as a fuel.
Sapotaceae	*Monotheca buxifolia* (Falk) A.DC. KUH-135	Gurgura	Tree	Whole plant	Fruit is Laxative, digestive and compensate iron deficiency. The fruit is also eaten. The plant is used as a fuel. It attracts honey bees. The plant is used for making agriculture tools.
Scrophulariaceae	*Kickxia ramosissma* Wall. KUH-130	Wangai	Herb	Whole plant	The plant is used as a fodder and fuel.
Solanaceae	*Datura innoxia* Mill. KUH-119	Randa	Shrub	Leaves	The leaves of plant are used as anti-inflammatory and laxative.
	*Solanum incannm* Linn. KUH-151	Marongay	Shrub	Leaves	Antidiabetic.
	*Solanum suretenses* Burn. KUH-152	Kandyari	Herb	Leaves and roots	Extract of plant is used as expectorant.
	*Withania coagulans* Dunal. KUH-155	Shopianga	Shrub	Fruit	Fruit is used for stomach pain.
	*Withania somnifera* (L) Dunal. KUH-156	Kwatilola	Shrub	Leaves and fruit	Fruit is given to children for removing abdominal pain. Green leaves are use forurinary tract inflammation.
Typhaceae	*Typha angustata* Bory. KUH-153	Dela	Shrub	Leaves	The plant is used for fuel purpose and thatching.
Verbenaceae	*Vitex negundo* Hausskn. KUH-154	Warmandai	Shrub	Leaves and root	Smoke of leaves is used as anodyne, leaves are boiled in oil and then used as wound healing. Roots are used for backache
Zygophyllaceae	*Fagonia cretica* Linn. KUH-125	Spelaghza	Herb	Whole plant	The extract of plant is used for diabetes mellitus and blood purification. It is also anti- inflammatory and anti-scabic.
	*Pegnum harmala* Linn. KUH-143	Sponda	Herb	Fruit and roots	Fresh fruit is used in pain of heart. Roots are used as lice- killing agent. It attracts honey bees.

**Table 2:** Average direct matrix ranking (DMR) score of fifteen key informants for ten medicinal plants species

**Use diversity**	**D. sisso**	**A. modesta**	**A. nilotica**	**C. desidua**	**M. boxifolia**	**Z. nummularia**	**M. nigra**	**M. azidarach**	**W. coagulans**	**R. stricta**	**Total**	**Rank**
Construction	5	2	3	1	2	3	4	3	1	1	25	2
Agriculture	4	2	2	3	2	2	2	1	1	1	20	5
Firewood	3	4	3	2	3	2	3	2	2	2	26	1
Fodder	2	3	3	2	2	3	3	2	3	1	24	3
Medicinal	1	3	3	3	2	2	1	2	3	3	23	4
Total	15	14	14	11	11	12	13	10	10	8		
Rank	1	2	2	5	5	4	3	6	6	7		
Market price in US$/Kg	0.6	0.5	0.5	0.2	0.53	0.4	0.4	0.2	0.9	0.8		

**Table 3:** **F**_**IC **_**values of traditional medicinal plants for treating human ailments in study area**

**Disease class**	**Nt**	**Nur**	**F**_**IC**_
Gastrointestinal	17	145	0.88
Respiratory	5	15	0.71
Wound healing	2	3	0.50
Dermatological	1	2	1.00
Kidney problems	2	4	0.66
Blood diseases	4	10	0.66
Cardiovascular	1	3	1.00
Inflammations	1	1	1.00
Hepatic problems	2	5	0.75
General pain	3	6	0.60
Fever	1	3	1.00
Dental	1	3	1.00

## Discussion

The study reveals that people of the area are much dependant on the native flora for acquiring their basic requirements such as medicines, fodder, fruits, vegetables, fuel, furniture and roof thatching. The local people utilize over 58 plants in their day to day life. All these species are the main sources of traditional health care needs and economic opportunity for the local people. Traditional healers of the study area mostly use herbs in the remedy preparation for the treatment of various ailments, which might be due to their strong therapeutic activity and easy extraction from the wild. Our study is in line with the findings of Mohammed [29], Gebre [30] and Teklehaymanot and Giday [31], who have enlisted various ethnomedicinal recipes being prepared from herb species. Family Solanaceae is the most dominant family in the study area, which mostly includes herbs and shrubs, play a vital economic role in the present study. The family is being used either as fuel wood or as medicinal species and are thus highly preferred.

The traditional healers and local herbalists of the region usually make use of every part of the plant. However, the use of a particular plant part depends on the plant habit and user’s needs. The most frequently used plant parts in the preparation of herbal remedies were leaves and whole plant. The use of specific plant parts suggests that these parts have strongest medicinal properties but it needs biochemical analysis and pharmaceutical screening to cross-check the local information. Our findings of the frequent use of green leaves in the preparation of remedies corroborate the results of [32-34]. Harvesting of whole plant for remedy preparation causes great danger to the plant population.

Main ailments in the study area are related to gastro-intestinal problems, respiratory infections and fever. The possible reason behind such ailments in the investigated region might be due to high water and air pollution, lack of proper sanitation, fuel wood smoke inside houses and poor quality food. Gastro-intestinal disorders and respiratory infections, particularly cholera, diarrhea and dysentery, cough, asthma and bronchitis are a major concern not only in the study area but the whole country and result in high mortality rate if not treated promptly [35]. These findings are similar to an ethnobotanical study conducted in the other parts of Pakistan, where species are locally used for related medicinal purposes [12,36]. Total seventeen species such as *Calotropis procera*, *Convolvulus arvensis*, *Capparis deciduas* and *Acacia nilotica* were found to be effective ethnomedicines for the treatment of gastrointestinal disorders. Murad *et al*. [37] also described the same plants using for the same ailment in Malakand region of Pakistan. Majority of the plants among them are used for the treatment of more than one type of ailment. This cultural similarity and multiple medicinal uses of single plant are the strong indication of availability of variety of therapeutic phytochemical. The highest recorded F_IC_ values (1.00, 0.88 and 0.71) indicated best agreement among informants’ on the use of medicinal plant species reported to be used for treating gastrointestinal, respiratory, fever, cardiovascular diseases respectively. The observed highest informants’ agreement coupled with high plant use citations for disease categories like gastrointestinal and respiratory infections could also indicate the relatively high incidence of the latter diseases in the area. According to Heinrich *et al*. [28], high F_IC_ values are important to identify plants of particular interest in the search for bioactive compounds. Plants use for disease categories such as fever, cardiovascular, dental and inflammatory infections recorded highest F_IC_ value and could be search for their bioactive compounds, however in comparison to gastrointestinal and respiratory infections these categories observed small number of plant use citation and less number of taxa. Tolossa *et al*. [38] also found gastrointestinal infection as one of the common ailment of the Ethiopian region.

The output of a DMR showed highest values (ranks) for a number of multipurpose medicinal plants of the study area such as *Dalbergia sisso*, *Acacia modesta*, *Acacia nilotica*, *Capparis desidua*, *Ziziphus nummularia* and *Monotheca buxifolia*. The result indicates that these plants are exploited more for their non-medicinal uses than for reported medicinal values. Overharvesting of multipurpose medicinal plant species for construction, fuel wood, fodder agricultural tools and other purposes were found the responsible factors aggravating depletion of the species in the area. Thus, the result calls for an urgent complementary conservation action to save the fast eroding multipurpose medicinal plant species of the area. Same pattern of highest exploitation of multipurpose medicinal plants for uses other than their traditional importance has been found [39]. *Dalbergia sisso* is the most valued species in the study area and DMR. According to Khan and Khan [40] the wood of *Dalbergia sisso* is highly preferred fuelwood and timber species. The informants selected for direct matrix ranking also indicated that most of the people of the region are also involved in exporting timber of *Dalbergia sisso* to the industries located in other regions of Pakistan for generating their income apart from their doemestic use as furniture and fuel wood. A study conducted in Gujrat region Pakistan indicated that eighty percent of industrial furniture is being made from *Dalbergia sisso*[41]. Other species like *Acacia nilotica*, *Acacia modesta*, *Ziziphus nummularia*, *Capparis desidua* and *Monotheca buxifolia* are also the most frequently using species for other non timber forest products. Zaman and Khan [42], Shahid ullah [43] and Ahmad [44] also reported multipurpose uses of the aforementioned species. Our findings are also in line with the study by Barkat *et al.*[45] carried out in district Malakand. He found that in the absence of gas supply and other fuel types in the area, the local people extensively use tree species as fuel wood. Ejaz *et al.*[46] also recorded the similar results in their study conducted in new Muree, Pakistan. Species such as *Acacia modesta*, *Asphodelus tenuifolius*, *Capparis desidua*, *Peganum hermala*, *Zizyphus jujuba* are considered as wild honeybee attraction species [47]. Honey of *Zizyphus* species is the most famous among the locals of study area due to its taste and medicinal value. Fine quality of honey is exported to markets at the provincial and national levels. Overharvesting and increasing trade of medicinal plants has significantly reduced the availability of the medicinal plants in arid and semi-arid region [48]. The present study observed that a large numbers of plants like are over harvested for medicinal and other NTFPs uses. Agriculture and livestock raring in the study area are common activities also to support rural livelihood. Therefore grazing is posing another pressure on the flora of the region. As an example, animals’ trampling can make the soil compact that retard seed germination and seedlings growth [49]. Moreover, goats raring are common in the study area, which can cause serious damage to plants through browsing. It is thus imperative to take necessary steps to protect the forests from such practices and device plans for range land management in order to improve the status of local vegetation.

It was noted that ethnomedicinal knowledge is becoming restricted only to the elders, *Hakeem’s* (traditional practitioners) and *Pensaries* (local herb sellers); while young people are totally ignorant of this wealth. Advancement in science and technology has changed the social values and therefore, younger generation are transforming at a much faster rate into the new tradition. Medicinal plants knowledge is going to be obsolete because of the interference of modern cultural changes. It is therefore very important to document the native flora along with their ethnomedicinal recipes before extinction of the indigenous knowledge.

## Conclusion

It is concluded from the present study that the natives of the region are very much dependent on plant species for their health care needs, fuel wood and fodder. Due to financial constraints, changing life styles and unavailability of resources, the local medicinal flora is facing overexploitation from the local inhabitants. Over grazing, fodder collection, logging and medicinal plants collection are major threats to the vegetation of the studied area. Such practices have resulted in the vulnerability of multipurpose species such as *Acacia modesta* and *Dalbergia sisso*. Hence, certain precautionary measures (controlled grazing, reforestation, rangeland management etc.) need to be addressed for the protection of threatened species. Moreover, in-situ and ex-situ conservation methods should be practiced as long-term conservation programme. Conservation education may be extended to the local communities and their local technologies may be incorporated in developing plans. Community mobilization and involvement may be assured in natural conservation. Alternative resources should be explored to reduce indiscriminate use and cutting for fuel purposes. Horticulture crops especially fruits, off season vegetable and mushrooms culture may be extended for the economic uplift of the area and reducing pressure of fuel wood on the forests.

Moreover, there is an urgent need to document the traditional knowledge of the area, which is another step towards the conservation of local flora. The local community may be educated and trained for the collection and sustainable use of medicinal flora. Additionally, the native healers may also be encouraged to accurately inculcate their traditional knowledge to local community.

## Competing interest

The authors declare that they have no competing interest.

## Authors' contribution

WM, AU, MA, SW and AA designed the research project and provided comments on the draft manuscript. AT has analyzed the data and wrote the draft of manuscript. KU conducted the field work. All authors have approved the final manuscript.

## References

[B1] Bako SP Bakfur MJ John I Bala EI Ethnomedicinal and phytochemical profile of some savanna plant species in Nigeria Int J Bot 2005 1 147 150

[B2] Walter W Hamilton A The vital wealth of plants 1993 UK: Bates and Sons Ltd

[B3] Ullah R Hussain Z Iqbal Z Hussain J Khan UF Khan N Muhammad Z Ayaz Z Ahmad S Traditional uses of medicinal plants in Dara Adam Khel NWFP Pakistan J Med Plants Res 2010 17 1815 1821

[B4] Sandya B Thomas S Isabel W Ethnomedicinal plants used by the Valaiyan community of Piranmalai hills (reserved forest), Tamilnadu, India Afr J Tradit Complement 2006 3 104 114

[B5] Qureshi R Floristic and ethnobotanical study of Desert-Nara Region, Sindh 2004 Shah Abdul Latif University, Pakistan Research Repository 454

[B6] Malik HMA Anwar R, Haq N, Masood S Treatment through Herbs Medicinal Plantsof Pakistan 2001 21 23

[B7] Abbasi MA Khan AM Mushtaq A Qureshi R Arshad M Jahan S Zafari M Sultana S Ethnobotaincal study of wound healing herbs among the tribal communities in Northern Himalaya ranges district Abbottabad, Pakistan Pak J Bot 2010 6 3747 3753

[B8] Khan AA Role of conservation of medicinal and aromatic plants in the socioeconomic development of rural poor’s 2003 International Workshop on conservation and sustainable uses of medicinal and aromatic plants in Pakistan: Joint venture by WWF-P, MINFAL and Qarshi Industries Pvt. Ltd

[B9] Hocking GM Pakistan medicinal plants Qualitas Plantarium Ethnobotanical Material Vegetablies 1958 5 145 153 10.1007/BF01099867

[B10] Ibrar M Hussain F Sultan A Ethnobotanical studies on plant resources of Ranyal Hills, District Shangla, Pakistan Pak J Bot 2007 2 329 337

[B11] Shinwari ZK Gilani SS Akhlas M Sustainable Harvest of Medicinal Plants at Bar and Shinaki Valleys, Gilgit (Northern Pakistan) 2003 WWF-P, Gilgit: Consultancy Report

[B12] Khan N Ahmed M Ahmed A Shaukat SS Wahab M Ajaib M Siddiqui MF Nasir M Important medicinal plants of Chitral Gol National Park (Cgnp) Pakistan Pak J Bot 2011 2 797 809

[B13] Ayyanar M Lgnacimuthu S Traditional knowledge of Kani tribals in Kouthalai of Tirunelveli hills, Tamil Nadu, India J Ethnoparmacol 2005 102 246 255 10.1016/j.jep.2005.06.020 16054791

[B14] Kumar GP Kumar R Chaurasia OP Singh SB Current status and potential prospects of medicinal plant sector in trans-Himalayan Ladakh J Med Plant Res 2011 5 2929 2940

[B15] Ali SI The significance of flora with special reference to Pakistan Pak J Bot 2008 30 967 971

[B16] Shinwari ZK Medicinal plants research in Pakistan J Med Plant Res 2010 4 161 176

[B17] Shinwari ZK Gilani SS Sustainable harvest of medicinal plants at Bulashbar Nullah, Astore (Northern Pakistan) J Ethnopharmacol 2003 84 289 298 10.1016/S0378-8741(02)00333-1 12648828

[B18] Hamayun M Khan SA Kim HY Leechae IJ Traditional know-ledge and ex-situ conservation of some threatened medicinal Plants of Swat Kohistan Pak J Bot 2006 2 205 209

[B19] Sher H Some Medicinal and Economic plants of Mahodand 2002 Utror, Gabral valley Swat

[B20] Sher H Midrarullah AU Khan Z Khan U Hussain F Ahmad S Medicinal Plants of Udigram, District Swat, Pakistan Pak J For 2003 1 65 73

[B21] Ali H Qaisar M The Ethnobotany of Chitral Valley, Pakistan with particular reference to medicinal plants Pak J Bot 2009 4 2009 2041

[B22] Jabeen A Khan AM Ahmad M Zafar M Ahmad F Indigenous uses of economically important flora of Margallah hills national park, Islamabad, Pakistan Afr J Biotechnol 2009 5 763 784

[B23] Awan RM Iqbal Z Shah MS Jamal Z Jan G Afzal M Majid A Gul A Studies on traditional knowledge of economically important plants of Kaghan Valley, Mansehra District, Pakistan J Med Plant Res 2011 16 3958 3967

[B24] Martin GJ Ethnobotany: A methods manual 1995 London: Chapman and Hall

[B25] Ali SI Qaiser M Flora of Pakistan 2010 No 1–215 (1972–2010), Pakistan http://www.efloras.org/flora_page.aspx?flora_id=5

[B26] Cotton CM Ethnobotany: Principles and applications Chichester 1996 New York: John Wiley and Sons Ltd

[B27] Yineger H Kelbessa E Bekele T Lulekal E Ethnoveterinary medicinal plants at bale mountains national park, Ethiopia J Ethnopharmacol 2007 112 55 70 10.1016/j.jep.2007.02.001 17368989

[B28] Heinrich M Ankli A Frei B Weimann C Sticher O Medicinal plants in Mexico: Healers’ consensus and cultural importance Soc Sci Med 1998 47 1859 1871 10.1016/S0277-9536(98)00181-6 9877354

[B29] Mohammed HA Traditional Use, Management and Conservation of Useful Plants in Dry Land Parts of North Shewa Zone of the Amhara National Region M.Sc. thesis 2004 Ethiopia: Addis Ababa University

[B30] Gebre G Ethnobotanical Study of Medicinal Plants in the Konso special Woreda (SNNPR) 2005 Ethiopia. M.Sc. thesis: Addis Ababa University

[B31] Teklehaymanot T Gidey M Ethnobotanical study of medicinal plants used by people in Zegie Peninsula, Northwestern Ethiopia J Ethnobiol Ethnomed 2007 3 12 10.1186/1746-4269-3-12 17355645 PMC1852296

[B32] Hussain M Shah GM Khan MA Traditional medicinal and economic uses of Gymnosperms of Kaghan valley, Pakistan Ethnobotany leaflets 2006 USA: SIUC

[B33] Shinwari ZK Rehman M Watanabe T Yoshikawa Y Medicinal and aromatic plants of pakistan: a pictorial guide 2006 Kohat, Pakistan: Kohat University of Science and Technology

[B34] Muthu C Ayyanar M Raja N Ignacimuthu S Medicinal plants used by traditional healers in Kancheepuram District of Tamil Nadu, India J Ethnobiol Ethnomed 2006 2 43 10.1186/1746-4269-2-43 17026769 PMC1615867

[B35] Ribeiro A Romeiras MM Tavares J Faria MT Ethnobotanical survey in Canhane village, district of Massingir: Mozambique: medicinal plants and traditional knowledge J Ethnobiol Ethnomed 2010 6 33 10.1186/1746-4269-6-33 21129187 PMC3016261

[B36] Adnan M Begum S Latif A Tareen AM Lee LJ Medicinal plants and their uses in selected temperate zones of Pakistani Hindukush- Himalaya J Med Plant Res 2012 6 4113 4127

[B37] Murad W Ahmad A Gilani SA Khan MA Indigenous knowledge and folk use of medicinal plants by the tribal communities of Hazar Nao Forest, Malakand District, North Pakistan J Med Plant Res 2011 7 1072 1086

[B38] Tolossa K Debela E Athanasiadou A Tolera A Ganga G Ethnomedicinal study of plants used for treatment of human and livestock ailments by traditional healers in South Omo, Southern, Ethiopia J Ethnobiol Ethnomed 2013 9 32 10.1186/1746-4269-9-32 23680260 PMC3679849

[B39] Lulekal E Asfaw Z Kelbessa E Damme PV Ethnomedicinal study of plants used for human ailments in Ankober District, North Shewa Zone, Amhara Region, Ethiopia J Ethnobiol Ethnomed 2013 9 63 10.1186/1746-4269-9-63 23984919 PMC3846447

[B40] Khan MM Khan MH Die-Back of Dalbergia sissoo in Pakistan 2010 II Faisalabad: University of Agriculture 921

[B41] Saleem A Siddiqui T Khan R Shisham wood consumption in furniture industry of Gujrat City Int J Agr Biol 2004 06 715 717

[B42] Zaman MB Khan SM Hundred drug plants of west Pakistan 1970 Pakistan Forest Institute Peshawar: Medicinal plants branch

[B43] Shahid ullah Ethnobotanical Studies of District Bannu, NWFP M.Phil Thesis 2000 Pakistan: Department of Biological Sciences, Quaid-e-University Islamabad

[B44] Ahmad M Ethnobotanical and Taxonomic study of economically important plants of Tehsil Attock (Distt. Attock) M.Phil Thesis 2003 Pakistan: Department of Biological Sciences, Quaid-i-Azam University Islamabad

[B45] Barkatullah V Ibrar M Hussain F Ethnobotanical studies of plants of Charkotli Hills, Batkhela District, Malakand, Pakistan Front Biol China 2009 4 539 548 10.1007/s11515-009-0045-2

[B46] Ahmed E Arshad M Saboor A Qureshi R Mustafa G Sadiq S Chaudary KS Ethnobotanical appraisal and medicinal use of plants in Patriata, New Murree, evidence from Pakistan J Ethnobiol Ethnomed 2013 9 13 10.1186/1746-4269-9-13 23445756 PMC3599915

[B47] Shahid M Qayyum A Bee Flora of the N.W.F.P Pakistan J Forestry P.F.I. Peshawar 1997 1 1 9

[B48] Singh V Pandey RP Medicinal plant lore of the tribals of East Rajasthan J Econ Taxonomic Botany 1980 1 137 147

[B49] Bravo ND Araujo MB Romdal T Rahbek C Scale effect and human impact on the elevational species richness gradients Nature 2008 453 216 220 10.1038/nature06812 18464741

